# A New Trend in Cancer Treatment: The Combination of Epigenetics and Immunotherapy

**DOI:** 10.3389/fimmu.2022.809761

**Published:** 2022-01-24

**Authors:** Zaoqu Liu, Yuqing Ren, Siyuan Weng, Hui Xu, Lifeng Li, Xinwei Han

**Affiliations:** ^1^ Department of Interventional Radiology, The First Affiliated Hospital of Zhengzhou University, Zhengzhou, China; ^2^ Interventional Institute of Zhengzhou University, Zhengzhou, China; ^3^ Interventional Treatment and Clinical Research Center of Henan Province, Zhengzhou, China; ^4^ Department of Respiratory and Critical Care Medicine, The First Affiliated Hospital of Zhengzhou University, Zhengzhou, China; ^5^ Internet Medical and System Applications of National Engineering Laboratory, Zhengzhou, China; ^6^ Medical School, Huanghe Science and Technology University, Zhengzhou, China

**Keywords:** epigenetics, immunotherapy, epigenetic regulation, T cells, immune checkpoint therapy, cancer therapy

## Abstract

In recent years, immunotherapy has become a hot spot in the treatment of tumors. As an emerging treatment, it solves many problems in traditional cancer treatment and has now become the main method for cancer treatment. Although immunotherapy is promising, most patients do not respond to treatment or develop resistance. Therefore, in order to achieve a better therapeutic effect, combination therapy has emerged. The combination of immune checkpoint inhibition and epigenetic therapy is one such strategy. In this review, we summarize the current understanding of the key mechanisms of how epigenetic mechanisms affect cancer immune responses and reveal the key role of epigenetic processes in regulating immune cell function and mediating anti-tumor immunity. In addition, we highlight the outlook of combined epigenetic and immune regimens, particularly the combination of immune checkpoint blockade with epigenetic agents, to address the limitations of immunotherapy alone.

## Cancer Immunotherapy

With technological advances in cell manufacturing and genetic engineering, as well as advances in immunology, molecular biology, and virology, immune cell therapy has been rapidly developed. Since the cellular division of immunological properties was defined, the function of adaptive immunity of B and T cells has attracted much attention (1). T cells have subsequently been demonstrated to have the ability to kill malignant cells, and the human immune system can eliminate cancer cells through acquired immune responses executed by T cells, which suggests that T cells can be rationally designed to control tumor growth. An increasing number of treatment modalities revolve around T cells to carry out research. Immunotherapy based on T cells is now regarded as an integral part of cancer treatment. However, clearing tumor cells by the immune system is not a simple process, which requires a series of conditions (2). First, cell death tumor-associated antigens are released from tumor cells into the tumor microenvironment to be captured by antigen-presenting cell (APC). Antigen-loaded APCs then process and present antigens along with major histocompatibility complex (MHC) complexes to the cell surface and transport them to lymphoid organs. Primitive T cells in lymphoid organs recognize selected peptide-MHC complexes through the T cell receptor (TCR), which triggers the priming and activation of effector T cells. Subsequently, differentiated effector T cells leave lymphoid organs to infiltrate into tumors through the circulatory system. T cells recognize cancer cells carrying matching antigens through TCR interaction with peptide-MHC complexes and kill cancer cells by direct or indirect immune attack. Immune attack leads to the release of additional antigens from dead tumor cells, which triggers a new round of anti-tumor immune response. However, tumor generation often develops by immune escape through various mechanisms due to failure of immune surveillance. For example, if there is a lack of APCs, APCs are inhibited or immune checkpoints are activated, these result in impaired capture of antigens released into the tumor microenvironment, which cannot mediate T cell priming and activation (3, 4). When T cells migrate or infiltrate into tumor tissue, they may not be performed due to the lack of appropriate chemokines and immunosuppressive tumor microenvironment (TME) (5, 6). The tumoricidal activity of T cells can also be blocked by regulatory cells in the TME (such as, regulatory T cells, macrophages, myelosuppressive cells, etc.), or by activating immune checkpoints on tumor cells or macrophages (7). In conclusion, the occurrence of any of the above conditions can lead to immune escape and thus bring about the generation of tumors. Therefore, immunotherapy has emerged to relieve immunosuppression and restore anti-tumor immune responses, which include immune checkpoint blockade therapy, adoptive cellular immunotherapy (8, 9), cytokine-based therapy (10, 11), and vaccines (12). The most remarkable of these is immune checkpoint blocking therapy (ICBT) against immune checkpoints. In March 2011, immune checkpoint inhibition was introduced as a new cancer therapeutic paradigm with FDA approval of the anti-cytotoxic T lymphocyte-associated antigen-4(CTLA-4) antibody ipilimumab for the treatment of advanced melanoma. Since then, inhibitors against the CTLA-4 and PD-1 immune checkpoints have revolutionized the treatment of not only melanoma, but also malignant tumors such as non-small cell lung cancer (NSCLC), renal cell carcinoma (RCC), and Hodgkin’s lymphoma. Arguably, the success of ICBT is the most significant advance in the field of cancer treatment in the past decade.

Immune checkpoint activation, which is the interaction of receptors between T cells and on tumor cells (13–15) and APCs (16–18). Currently the most extensively studied are PD-1 and CTLA-4, as well as their respective ligands PD-L1 and CD80 or CD86. Therefore, the basic principle of immune checkpoint inhibition is to use antibodies against PD-1, PD-L1, or CTLA-4 for treatment to reverse the inhibitory effect of immune checkpoints and promote anti-tumor effects by preventing the interaction of these receptors. However, the clinical response of immune checkpoints depends on the immune status of the tumor. The presence of antigen-specific CD8^+^ lymphocytes within the TME is a primary condition (19–21). Second, the composition of nearby immune cell populations must differentiate into an immune-permissive state (22–25). Third, tumors must have MHC class I-mediated antigen presentation functions (26). Only tumors with these characteristics can receive immune attack, otherwise it will be a state of immune evasion (27, 28), which allows cancer to evade immune detection and grow freely. These conditions make ICBT clinically limited, and most patients do not respond to treatment or develop resistance. Therefore, it is necessary to find a new mode of immunotherapy to overcome the dilemma, and the combination of immune checkpoint inhibition and epigenetic therapy is one such strategy. It has been shown that epigenetics can improve immune recognition and immunogenicity and thus play an important role in immune evasion (29–31). Although the concept of cooperation between epigenetic therapy and strategies such as immune checkpoint therapy has only recently emerged, many studies have highlighted the potential of this combination approach in many different cancer types (31–34). In addition, some ongoing clinical trials are currently exploring the effectiveness of this combination approach. In this review, we address the current understanding of the key mechanisms of how epigenetic mechanism influences cancer immune responses and reveal the key role of epigenetic processes in regulating immune cell function and mediating anti-tumor immunity. In addition, we highlight the outlook of combined epigenetic and immune regimens, particularly the combination of immune checkpoint blockade with epigenetic drugs, to address the limitations of immunotherapy alone.

## Role of Epigenetics in Cancer Therapy

Epigenetic dysregulation is a major mechanism in cancer development and progression (35, 36). Epigenetic regulation is a DNA-heritable modification that alters chromatin structure and gene expression without altering the underlying nucleotide sequence (37, 38). The modification process is mainly through changing the three-dimensional distribution of nucleosomes throughout the genome so that the way DNA is packaged is changed. This packaging process is fine-tuned by covalent labeling of amino acids on histones in the context of nucleosomes and methylation-mediated interactions of genomic DNA at CpG sites (38–40). In addition to DNA methylation, histone post-translational modifications, such as acetylation, methylation, and generalization, are also key regulators of chromatin structure that affect gene expression. There are also a variety of mechanisms that regulate the transcriptional state of genes: chromatin remodeling; histone variant exchange; and the role of non-coding RNAs. Epigenetic modifications of DNA and histones dynamically and reversibly regulate transcription, allowing chromatin to interconvert in both closed (heterochromatin) and open (euchromatin) states. The chromatin structure in the open state can allow access of transcriptional activators such as RNA polymerase and DNA-binding transcription factors to target genes and promote active transcription. In contrast, closed state chromatin is usually associated with transcriptional silencing (41). Over the past few decades, attention has been paid to the development of epigenetic therapies as anticancer agents based on their direct effects on cancer cells. While recent studies have elucidated how epigenetic mechanisms acts on immune evasion, they have revealed the role of epigenetic drugs in modulating immune pathways to improve immune recognition and immunogenicity (29–31). A full understanding of the role of epigenetic regulatory mechanisms in cancer immunity is essential to exploit the potential of epigenetic drugs.

### Epigenetic Alterations in Tumor Cells

Aberrant DNA methylation may be an important event leading to tumor development. In the 1980s, hypomethylation of genome-wide DNA was first observed in cancer cells (42), which may cause genomic instability, chromatin structure changes, as well as some gene expression rises. The specific methylation level of the gene promoter showed an elevated state. Aberrant methylation patterns are often associated with frequent mutations in genes that regulate DNA methylation (such as DNMT3a and TET2) in human cancers, leading to abnormal gene expression in human cancers. For example, local hypermethylation of tumor suppressor gene promoters silences their expression, which is directly associated with tumorigenesis (43). Abnormal patterns of histone modifications are also common in tumor cells. The number of modifications and modifications at different sites of histones is of great interest for transcriptional regulation of genes in tumor cells. For example, H3K4me3, which is widely studied, mediates the activation of transcription. On the other hand, H4K20me3 is closely related to the silencing of repetitive DNA and transposons (44) and mediates transcriptional repression (45). Loss of H4K20me3 is considered an important feature of cancer (46). Histone H4K16 acetylation and loss of H4K20 trimethylation have been reported as common hallmarks of human cancer (46). Post-translational modifications of histones together with DNA methylation determine the fate of gene expression which leads to the development of tumors. Moreover, epigenetics also affect anti-tumor immune responses, such as inducing neoantigen production, disrupting antigen presentation mechanisms, promoting inflammatory factor production and inducing immunosuppressive effects, thereby exacerbating tumor development.

Epigenetic alterations may lead to the reactivation of genes, which brings about the formation of new antigens in most cancers (47). The most typical example is the generation of Cancer/testis antigens (CTAs). CTA is an ideal target for cancer immunotherapy, especially for cancer vaccines and adoptive cell therapy, which is encoded by a set of genes that are mainly expressed in male germ cells under healthy conditions (48). However, CpG demethylation associated with these genes, as well as other epigenetic dysregulations, can re-express the gene encoding CTA in tumors. When CTAs, protein products of these genes, are reactivated in tumor tissues without immune privilege, they can induce adaptive immune responses, whose strong immunogenicity and tumor specificity make it a priority target for cancer immunotherapy (49).

Epigenetics can also cause dysregulation of antigen presentation mechanisms in tumor cells, making T cells unable to effectively recognize tumor cells. The presentation of tumor antigens requires the expression of MHC class I on the cell surface, which can be inhibited by DNA methyltransferase enzymes (DNMT) and histone deacetylase (HDAC). It has been demonstrated by the re-expression of MHC class I after DNMTi and HDACi treatment of cells (50, 51). Treatment of tumor cells and patients with DNMTi results in increased expression of genes required for antigen presentation (52). Histone methylation is also an important epigenetic mechanism leading to silencing of immunogenic factor expression, and its most obvious role is to inhibit MHC class I antigen presentation. In SCLC and neuroblastoma, targeted inhibition of histone methyltransferases can upregulate the expression of MHC class I in tumor cell lines. Similar findings have been observed in lymphomas (53).

**Figure 1:**
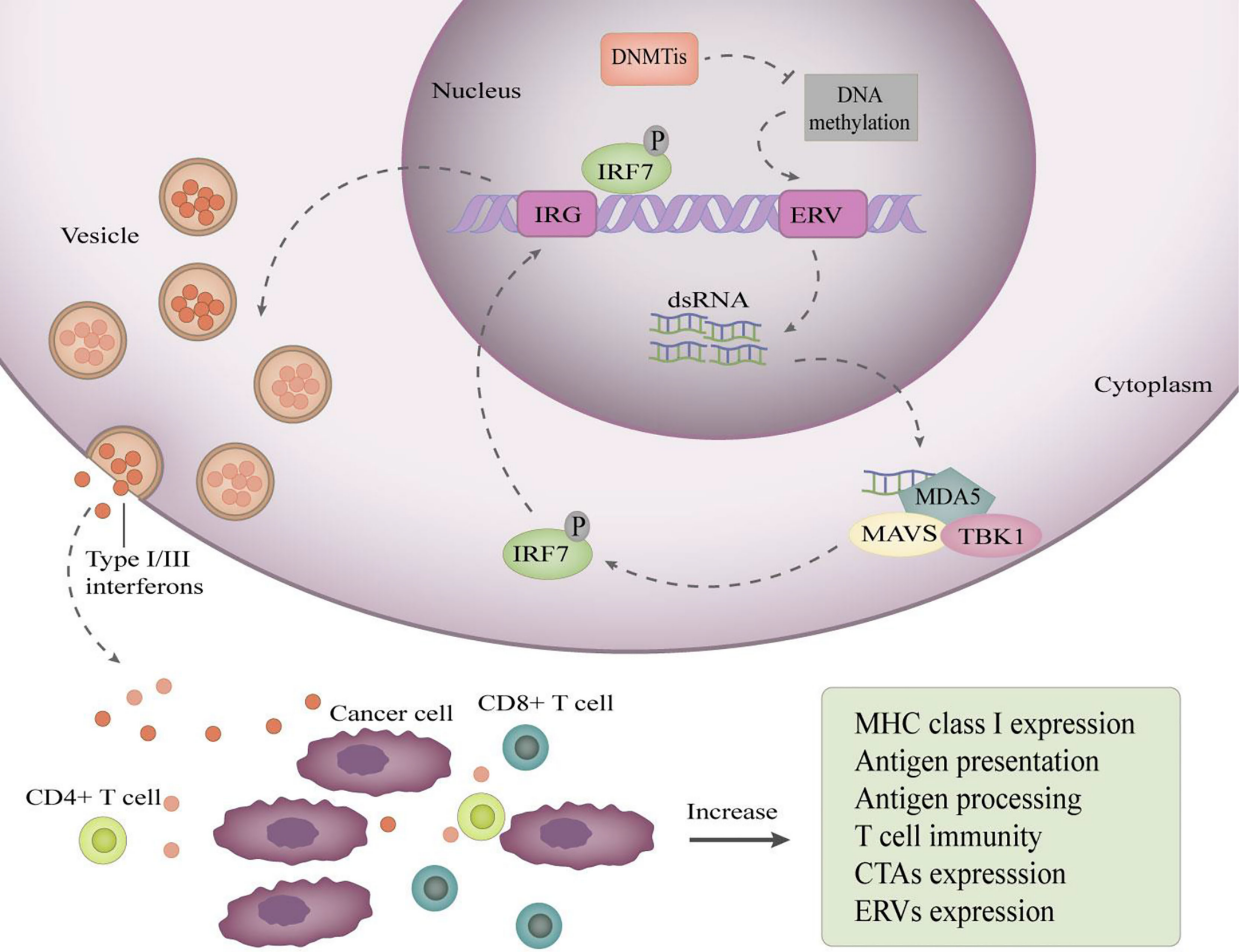
DNA demethylation restores ERV expression to induce viral mimicry. DNA demethylating drugs reactivate ERV promoters by inhibiting their methylation, resulting in bidirectional transcription of ERVs to produce dsRNAs, which are exported to the cytoplasm and sensed by pattern recognition receptors, such as MDA5. MDA5 binding to dsRNA induces recruitment of TANK-binding kinase 1 (TBK1) and aggregation of mitochondrial antiviral signaling protein (MAVs), which activate interferon regulatory factor 7(IRF7) by phosphorylation. Then, activated IRF7 moves into the nucleus and induces transcription of interferon-responsive genes (IRG). Consequently, type I/III interferons are produced, transported, and secreted into the tumor microenvironment. Secreted type I/III interferons increase the expression of antigen processing and antigen presentation mechanisms, improving the ability of cancer cells to present antigens.

Inflammatory cytokines are essential for the immune system. The differentiation, activation, entry of immune cells and immune attack on tumor cells are inseparable from inflammatory cytokines. Epigenetic mechanisms can regulate specific genes to promote the production of proinflammatory cytokines in tumor cells. Endogenous retroviruses (ERVs) are transposon elements in the genome that are silenced by DNA methylation in the human genome. ERV promoter DNA demethylation restores ERV expression. Activation of ERV brings about a “viral mimicry” state (54), in which tumor cells behave like virus-infected cells and initiate an innate immune response, leading to the production of type I and type III interferons (54, 55) (**Figure 1**). Autocrine and paracrine type I interferon signaling in TME promotes the production of proinflammatory cytokines and chemokines, resulting in enhanced tumor cell immunogenicity, and these changes can improve the effectiveness of immune checkpoint inhibitors (54, 55).

Tumor-induced immunosuppressive effect is one of the main reasons for tumor immune escape, and tumor-produced immunosuppressive molecules, such as PD-L1, can directly inhibit the immune response as well as recruit regulatory T cells that secrete immunosuppressive cytokines by themselves. Epigenetic mechanisms contribute to the regulation of PD-L1 expression, such that it is upregulated in tumor cells. For example, in glioblastoma multiforme and prostate cancer, PD-L1 expression and prognosis are inversely correlated with methylation of the PD-L1 gene promoter (56, 57). Studies have shown that in patients with metastatic melanoma treated with anti-PD-1 therapy, circulating exosome PD-L1 levels are positively correlated with interferon-γ (IFN-γ) signaling, which can stimulate PD-L1 expression (58). Inhibition of BET protein, the reader of histone acetylation, inhibits IFN-γ-induced PD-L1 expression (59, 60) In addition, in mouse models of ovarian cancer, BET inhibitors can reduce PD-L1 expression in tumor cells, tumor-associated dendritic cells and macrophages, thereby limiting tumor progression (61).

### Epigenetic Effects on Immune Cells

Over the past decade, some studies have shown that the fate of cell differentiation during lymphocyte development is largely influenced by epigenetic mechanisms (62). To some extent, epigenetic mechanisms can determine the functional and phenotypic changes of cells during activation of the adaptive immune system. For example, the function of dendritic cell (DC) is regulated by chromatin structure and histones. It has been shown that the activation of bone marrow-derived DCs is inhibited by the histone-H3K4-specific demethylase KDM5B, resulting in T cell responses that cannot proceed normally (63). Epigenetic regulation of cell differentiation has been studied in several major immune cell populations, including CD8^+^ T cells, CD4^+^ T cells, and myeloid cells.

Naive CD8 T cell responses in lymph nodes require the initiation of an autonomous program of differentiation and proliferation, which is the result of stimulation after antigen presentation by specialized antigen-presenting cells. During these processes, the epigenetic landscape of T cells changes (64). Under acute stimulation, naive T cell proliferates and differentiates into effector T cells to remove antigens. After antigen removal, a small proportion of memory effector cells survive the immune response stage and develop into functional memory T cells. Consequently, functional memory T cells can rapidly differentiate into effector T cells to perform immune effector function when they meet antigens again. However, under continuous antigen stimulation, the sensitivity of T cells to antigen response is reduced, and finally effector response cannot be produced to achieve a state of nonfunctional differentiation, which is called T cell depletion. Epigenetic programs influence each of these stages of differentiation. Gains and losses in genome-wide DNA methylation and histone modifications were observed during the differentiation of primitive CD8^+^ T cells into CD8^+^ effector T cells (65–67) The production of key effector genes by antigen-stimulated naive CD8^+^ T cells, as well as the transcription start site (TSS) of transcription factors expressed in activated lymphocytes are demethylated, while genes associated with naive T cells, such as CCR7 and Tcf7, evolve T cell differentiation by increased methylation of the TSS to promote gene silencing (68, 69). Similarly, epigenetic mechanisms also regulate CD8^+^ effector T cell dedifferentiation into memory T cells (70, 71). Memory-precursor CD8 T cells complete the reversal of epigenetic suppression of naive T cell-associated genes by demethylating key genes expressed in CD8^+^ effector T cells (66, 70). It has been demonstrated that the DNA methylase DNMT3a is involved in inhibiting memory CD8 T cell production (70). Epigenetic mechanisms are responsible for T cell exhaustion as well (66, 72). HDAC inhibitors can reverse the functional status of T cell exhaustion (73).

Regulatory T cells (Treg) associated with cancer progression that come from the transformation of traditional CD4^+^ T cells have the ability to suppress immune responses and accumulate in both animal models and cancer patients (74). The growth and development of Treg cells are tightly regulated by epigenetics. EZH2 histone methylases deposit H3K27me3 marks in the regulatory elements of genes down-regulated in Treg cells in order to regulate the development of Treg cells (75, 76). EZH2 inhibition may prevent the accumulation of Treg cells in cancer thereby relieving their suppression of immune responses. Epigenetics also controls the expression and activity of Treg cell-specific genes, including Foxp3, a key transcription factor used to identify Treg cells (77). The gene encoding Foxp3, which controls development and function of Treg cells, is usually methylated (78–80), and silenced in naïve T cells or activated CD4^+^ T cells, but methylated and expressed in Tregs (81). Foxp3 protein promotes Treg development through acetylation of HDAC9. Thus, effector differentiation of CD4 T helper cell lines is plastic and can be reversed in response to appropriate environmental stimuli with the participation of dynamic changes in epigenetics and transcription (82). The transcriptionally active mark H3K4me3 can be found at the locus of cytokine genes unique to each TH, while the repressed H3K27me3 mark turns other genes off (83).

Myeloid-derived suppressor cells (MDSCs) are a cell population known to induce peripheral blood T cell tolerance and inhibit T cell activation and proliferation (84–86), whose fate is also modulated by epigenetic modifications. Differentiation and activation of MDSCs mainly involves various histone modifications that regulate the binding of specific transcription factors to their target genes mainly by keeping the chromatin structure in an open state (87, 88). Sahakian et al. found that knockdown of histone deacetylase 11 (HDAC11) gene showed more inhibition of MDSC number in a mouse tumor model, suggesting that MDSC expansion and function is negatively regulated by HDAC11 (89). Zhang et al. also demonstrated that in addition to DNA methylation and histone acetylation, miRNAs and siRNAs can also eliminate cancer cells by altering the properties of MDSCs (90).

## Metabolic Dysregulations Are Linked to Epigenetic Changes in Cancer and Immune Cells

Previously, there were limitations in our understanding of cancer, and it was believed that from tumor initiation, growth to metastasis, they were dominated by genetic mutations. In recent years, cellular metabolic remodeling and epigenetics, as one of the characteristics of cancer, are gradually well-known for the importance of tumor development. Tumor cells will show tightly regulated metabolic plasticity during tumorigenesis and metastasis. Like tumor cells, cellular metabolism is also a key factor in the maintenance of viability and function of immune cells. The advent of immunotherapy has made it increasingly important to understand more about the metabolic relationship between infiltrating tumor cells and immune cells. It has been shown that certain metabolic changes occur at the epigenetic level, and that many metabolites can act as substrates or cofactors for chromatin-modifying enzymes, which closely link epigenetics and metabolism and regulate each other. In some cases, various metabolic alterations and epigenetic modifications can prompt impeding immune surveillance or immune escape, thus playing an important role in tumor progression.

The most important cellular mechanism affecting the epigenetic landscape of tumor cells is the reprogramming of metabolic pathways, during which the characteristics of metabolites are changed (91, 92), producing the main players and regulators of epigenetic modifications. Accumulating evidence suggests that cellular intermediate metabolites drive the expression of epigenetic mechanisms through chemical post-translational modifications that alter chromatin structure and function (93, 94). The intertwined relationship between epigenetic modifications and metabolomes plays a very important role in the development and progression of tumor cells. First, metabolites in tumor cells affect the epigenetic modification landscape as cofactors of modification enzymes., modification donors, or antagonistic molecules. Almost all epigenetic modification processes require the participation of metabolites. acetyl-CoA produced from glycolysis, NAD+ produced from the combination of glycolysis and oxidative phosphorylation, and S-adenosyl methionine (SAM) generated from a carbon cycle as a substrate or cofactor involved in DNA methylation and posttranslational modification processes of histone (95). Moreover, metabolic enzymes also have a great impact on the regulation of epigenetics. For example, DNMT mediates DNA methylation using SAM as a methyl donor, and histone methylation catalyzed by histone methyltransferase (HMT) also requires the participation of SAM (96). The metabolic enzyme nicotinamide N-methyltransferase (NNMT) can catalyze the transfer of the methyl moiety from SAM to nicotinamide, thereby decomposing SAM into 1-Methyl Nicotinamide (1MNA). Cancer cells overexpressing NNMT have shown alterations in their SAM and histone methylation levels while acquiring a more aggressive phenotype (97). The reaction catalyzed by NNMT hinders the SAM mediated DNA and histone methylation process. Therefore, metabolites and metabolic enzymes play a very wide and important role in epigenetic modification of tumors. Second, epigenetic modifications can directly alter the expression of metabolic enzymes and transporters or regulate cellular metabolism by affecting the expression of signal transducers and transcription factors. For example, the hypomethylation state of genomic DNA allows the expression of PKM2, the rate-limiting enzyme of glycolysis, to be up-regulated in a variety of tumors (98).

Metabolic reprogramming of cancer cells has emerged as a key immunosuppressive mechanism to modulate anti-tumor immune responses. Metabolic status plays multiple roles in determining innate immune cell function and fate (99). Infiltrating CD8^+^ T cell metabolism in the tumor microenvironment is often characterized by functional disorders and unique epigenetic manifestations in tumors or other tissues (100), which are all major factors affecting anti-tumor immunotherapy. It has been reported that tumor cells can affect epigenetic modification of T cells by regulating metabolites in their microenvironment. Tumor cells disrupt methionine metabolism in CD8^+^ T cells, thereby reducing intracellular levels of methionine and the methyl donor SAM and leading to loss of dimethylation at lysine 79 of histone H3 (H3K79me2), which leads to low expression of STAT5 and impaired T cell immunity (101). Since T cell function requires activation of many metabolic pathways to provide energy and raw materials, metabolic reprogramming is essential for T cell activation and differentiation. Among them, polyamine synthesis is a marker of T cell activation and proliferation. Puleston et al. reported that polyamine-hypusine deficiency leads to extensive epigenetic remodeling driven by altered histone acetylation and a re-wired tricarboxylic acid (TCA) cycle, which has an impact on the ability of CD4^+^ helper T cells to differentiate into different functional fates (102). Accumulating evidence suggests that metabolism affects cell signaling and epigenetics, thereby controlling the lifespan of T cells and converting T cells to an exhaustion state, which inhibits effector function and leads to adverse effects on immune checkpoint molecules (ICM) targeted therapies. How metabolic stress affects T cell exhaustion remains an active area of research (103).

## Epigenetic Drugs Enhance Anti-Tumor Immune Responses

The ability of epigenetic drugs to upregulate the expression of immune signaling components in cancer cells has been established (29, 34, 104), such as histone deacetylase inhibitors (HDACi) and DNA methyltransferase inhibitor (DNMTi). DNMTi, commonly known as demethylating agents, is the most widely used epigenetic therapy for the treatment of cancer. They are analogues of nucleoside cytidine that irreversibly sequester DNMT proteins from DNA, leading to global DNA hypomethylation. HDACis interfere with the function of histone deacetylases and act by controlling the degree of tightness of DNA wrapped around histones. Treatment of affected tumor animals with DNMTi and/or HDACi can alter immunosuppressive TME and enhance tumor-infiltrating lymphocytes (50, 105–107). These effects are the result of enhanced tumor antigen expression and/or presentation, “viral mimicry” effects, inhibition of T-cell exhaustion, induction of chemokine expression, or a combination thereof.

The methylation effect of DNMTis can lead to CTA re-expression in cancer cells of many different solid tumors (108–110). And 5-Azacytidine can increases the anti-tumor T cell profile in patients with Hodgkin’s lymphoma, suggesting that inhibition of DNMT improves new antigen presentation capacity and immunogenicity in tumor cells. In addition to CTA, other TAAs are also regulated by epigenetic drugs, such as high molecular melanoma-associated antigens (HMW-MAAs). 5-AZA-CdR demethylates the HMW-MAAs gene promoter in melanoma cells, resulting in the re-expression of HMW-MAAs at the mRNA and protein levels (111). Although the induction of CTA up-regulation by HDACi is much lower than that by DNMTis (112), it can induce the expression of MHC class I to increase antigen presentation. In the mouse melanoma model, inhibition of HDAC-I with romidepsin enhanced MHC- I expression and enhanced killing activity of CD8^+^ T cells. Moreover, HDAC inhibition also induces the expression of MHC class I antigen processing and presentation genes, including TAP1, TAP2, LMP2, LMP7 and B2M (113–115).

A key pathway by which DNMTi upregulates immune signaling in cancer is through the viral mimicry pathway. In ovarian cancer cell lines, DNMTis promotes transcription of dsRNA by repressing the silent expression of hypermethylated endogenous retroviruses (ERVs), upregulates dsRNA activates cytoplasmic dsRNA sensors and activates downstream signaling pathways, and induces IFN-β signaling (55). The production of type I and type III interferons induced by the viral mimicry pathway would increase antigen presentation and processing of cancer cells in the tumor microenvironment. Roulois et al. had similar findings in colon cancer cells treated with 5-AZA-CdR (54). The ERVs represent a large fraction of repetitive elements in the human genome that are silenced by DNA methylation. Treatment with DNMT inhibitors allows cancer cells to enter a “viral mimicry” state in which they behave like virus-infected cells, leading to activation of the interferon pathway. These changes were shown to enhance the effectiveness of immune checkpoint inhibitors (54, 55). Further studies revealed that histone deacetylases (HDACs) and KDM1A, the “eraser” of H3K4me1/2 also have similar effects in inhibiting ERV and ERV-induced interferon pathway activation (105, 116).

T cell exhaustion is one of the major causes of immune evasion. A state of T cell differentiation induced by continuous antigen stimulation, resulting in impaired cell function. It is characterized by reduced production of effector molecules and expression of multiple inhibitory receptors including PD-1 (117, 118). T cell exhaustion may be responsible for rendering patients treated with checkpoint inhibitors unresponsive or relapsing. Blockade of PD-1 can only partially and temporarily reverse the phenotype of these T cells that have undergone chronic stimulation with antigen, and epigenetic interventions may help revitalize exhausted T cells. Indeed, treatment of exhausted T cells with HDAC inhibitors restores their functional status (119). In a mouse model of melanoma, the combined use of anti-PD-1 and HDACi therapy was shown to improve survival in mice (120). In the context of chronic antigen exposure, DNMT3a performs methylation of a program associated with exhaustion in CD8^+^ T cells. Inhibition of DNMT3a reverses the phenomenon and prevents T cell exhaustion (66).

Epigenetically suppressed chemokines have recently been found to have an important role in tumor immune escape. These chemokines would protect tumor cells from immune responses, affecting immune cell infiltration of TME mainly by inhibiting the trafficking of T cells. In ovarian cancer, H3K27me3 and DNMT1 epigenetically regulate the Thelper1 (Th1) type chemokines CXCL9 and CXCL10, which determine their production (121). Epigenetic regulation using DNMTi is able to induce expression of chemokines and infiltration of Th1 tumors. In lung cancer, HDACis have also been shown to have similar effects that can enhance the expression of T cell chemokines and the infiltration of TME (65). In addition, epigenetic drugs can also increase immune-mediated cytolysis and tumor cell recognition through the action of the innate immune system. For example, HDACi treatment can increase the expression of NK cell surface activating receptor NKG2D by increasing the binding of H3 acetylation on gene promoters, thereby enhancing NK-mediated tumor cell targeting (122). Several different HDACIs have also been shown to increase NK cell killing of tumor cells by upregulating the stress-inducing ligands, such as MICA, MICB, and ULBP1-3, in tumor cells from many different solid malignancies (123–125).

## Combination Therapy of Epigenetic Drugs and Immune Checkpoint Inhibitors

**Figure 2:**
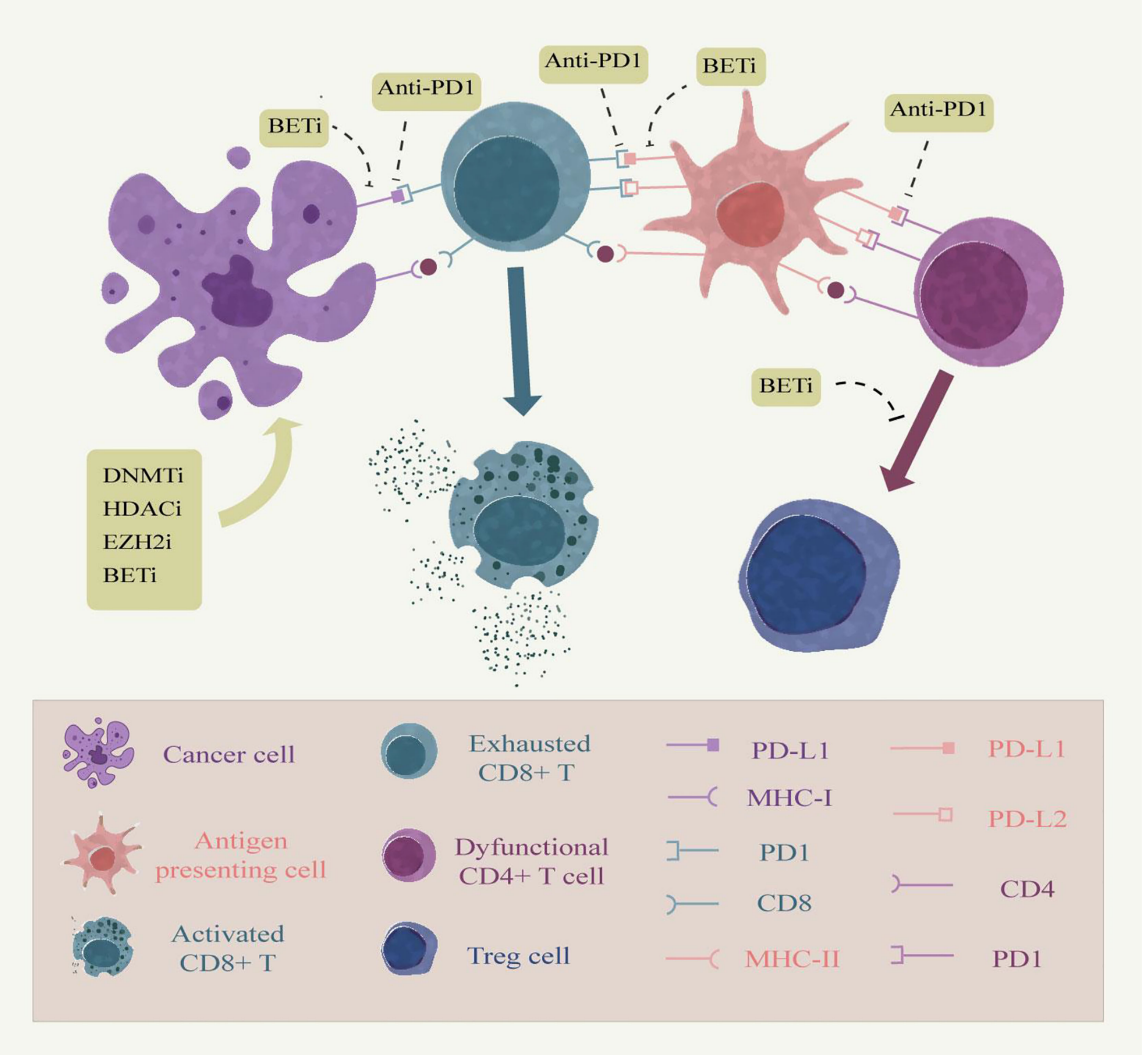
Combining epigenetic drugs with immune checkpoint inhibitors. Persistent antigen stimulation and inflammatory factors in chronic inflammation can cause dysfunction of tumor-infiltrating T cells, up-regulation of immune checkpoints, and production of immune evasion, which are associated with epigenetic modifications. Anti-PD-1 relieves the inhibitory effect of the epidemic checkpoint on tumor cells and antigen presenting cells by blocking the binding of PD-1 to its ligands PD-L1 and PD-L2 in the tumor microenvironment. Epigenetic modifiers can enhance antigen presentation by tumor cells, thereby enhancing the immune effects of T cells. Moreover, epigenetic modifiers inhibitors, such as BET, can also inhibit the expression of PD-L1 on the surface of tumor cells and tumor-infiltrating immune cells. Moreover, EZH2 inhibitors prevent the conversion of CD4 T cells into Treg cells to up-regulate the immune response of other cells. Therefore, the combination therapy of epigenetic modifiers with immune checkpoint inhibitors embodies great advantages.

As mentioned above, epigenetic mechanisms have an important impact on both host immune cells and tumor cells, and epigenetic drugs have been demonstrated to improve cancer immunotherapy efficacy in many aspects. The combination of immunotherapy and epigenetic drugs is an upsurge in the study of cancer treatment in recent years, the most remarkable of which is the combination of immune checkpoint blockade therapy and epigenetics (**Figure 2**). The classical epigenetic drugs HDACi and DNMTi have been approved by the FDA for cancer therapy. In the animal model of ovarian cancer, the addition of the demethylating drug azacitidine to anti-CTLA-4 antibody therapy significantly elevated the expression of chemokines by NK cells and CD8^+^ T cells, inhibited tumor growth and prolonged survival in ovarian cancer models compared with immune checkpoint inhibition alone (126). Other studies have provided evidence that the use of DNMT inhibitors can also enhance the effectiveness of anti-PD-1 antibodies. Yu et al. described the mechanism by which decitabine enhanced the expression of immune-related genes such as major histocompatibility complex genes and cytokine-related genes in a syngeneic mouse CT26 colon cancer model and found an increased accumulation of cytolytic CD8^+^ T cells in the tumor, demonstrating the sensitizing effect of decitabine against PD-1 antibody therapy (127). In addition, azacytidine was able to up-regulate the expression of PD-L1 gene at the transcriptional level and also directly on the cell surface in an *in vitro* cell lung cancer cell line model. Identifying the use of epigenetic therapies in checkpoint inhibitor therapy may elicit more potent immune responses (128).

The regulation of HDAC is multifaceted, which involves NK cell ligand activation and increased cytotoxicity, regulation of MHC class I and class II molecules, elevation of proinflammatory cytokines, and regulation of Treg and Treg Foxp3 gene expression (129). It has been shown that panobinostat is able to modulate different serum cytokines associated with T cell activation in patients with Hodgkin’s lymphoma while entinostat can induce immune-related genes associated with antigen presentation in breast cancer (130). PD-L1 expression of tumor antigen presenting cells and T cells was upregulated after treatment of various solid tumor arterial models with HDAC and CTLA-4 inhibitors. Inhibition of HDAC, PD-1, and CTLA-4 can lead to complete tumor rejection. In addition, the HDAC inhibitor entinostat induces depletion of MDSCs and enhances the efficacy of anti-PD-1 therapy (131, 132). Studies have shown that the up-regulation of immune checkpoints is epigenetically regulated through the action of HDACi that regulate PD-L1 expression in melanoma. In the mouse melanoma cell model, mice treated with a combination of panobinostat and anti-PD-1 showed slower tumor progression and higher survival (120).

In recent years, epigenetic drugs with new targets have also gradually entered the horizon of researchers and are approved for cancer treatment. In 2020, the EZH2 inhibitor, Tazverik, was approved for the treatment of epithelioid sarcoma, making it the first approved histone “writer” inhibitor and the first to be used to treat solid tumors (133). Goswami et al. found that peripheral blood T cells from patients treated with anti-CTLA-4 antibody increased EZH2 expression (121, 134). Subsequently, they demonstrated that EZH2 inhibitor alone enhanced the cytotoxic activity of human CD8^+^ effector T cells, altered the phenotype and function of human Treg cells, and had an immunotherapy-sensitizing effect against CTLA-4 in mouse bladder cancer and melanoma models (121, 134). In addition, the combination of EZH2 inhibitors and azacytidine increases immune cell infiltration in TME, slows tumor progression, and improves the efficacy of anti-PD-L1 therapy (121). Other drugs, such as inhibitors against LSD1, PRMT5, and BET proteins, can also enhance the efficacy of immunotherapy. Together, these findings provide evidence to support the effectiveness of combining epigenetic agents and immune checkpoint inhibition.

## Conclusion

The rapid development of cancer immunology has attracted a lot of research efforts and achieved outstanding results. Immune checkpoint blockade represents a new milestone in cancer therapy with promising prospects in terms of clinical benefit and enhanced durability of tumor response. Recent studies have shown that epigenetic regulation affects all aspects of the interaction between tumor cells and the immune system. Thus, epigenetic regulation can induce robust antitumor immune responses. The combination of epigenetic regulation and immunotherapy has been proved can relieve some of the limitations of single immunotherapy, which makes it a promising combination therapy partner for cancer immunotherapy. In this review, we have elucidated the mechanism of the immunological effects of epigenetic regulation on tumor cells and immune cells, and discussed the combination therapy of epigenetic drugs and immune blocking point inhibition therapy. There is an increasing number of epigenetically targeted drugs approved for cancer therapy, and their combination with immunotherapy will certainly have more possibilities. The future will also see the development of new methods that represent the combination of genetic drugs with emerging immunotherapies, including tumor vaccine and adoptive T cell therapies, which will face great challenges, but also provide new opportunities for improving cancer therapeutic interventions.

## Author Contributions

ZL designed this work. YR collected materials. YR wrote this manuscript. ZL, XH, SW, HX, and LL edited and revised the manuscript. All authors have read and agreed to the published version of the manuscript.

## Funding

This study was supported by the National Natural Science Foundation of China (Grant No. 82002433).

## Conflict of Interest

The authors declare that the research was conducted in the absence of any commercial or financial relationships that could be construed as a potential conflict of interest.

## Publisher’s Note

All claims expressed in this article are solely those of the authors and do not necessarily represent those of their affiliated organizations, or those of the publisher, the editors and the reviewers. Any product that may be evaluated in this article, or claim that may be made by its manufacturer, is not guaranteed or endorsed by the publisher.

## References

[1] Cooper MD Miller J. Discovery of 2 Distinctive Lineages of Lymphocytes, T Cells and B Cells, as the Basis of the Adaptive Immune System and Immunologic Function: 2019 Albert Lasker Basic Medical Research Award. JAMA (2019) 322:1247–8. doi: 10.1001/jama.2019.13815 31503279

[2] Chen DS Mellman I. Oncology Meets Immunology: The Cancer-Immunity Cycle. Immunity (2013) 39:1–10. doi: 10.1016/j.immuni.2013.07.012 23890059

[3] Fu C Jiang A. Dendritic Cells and CD8 T Cell Immunity in Tumor Microenvironment. Front Immunol (2018) 9:3059. doi: 10.3389/fimmu.2018.03059 30619378 PMC6306491

[4] Zhang Y Du X Liu M Tang F Zhang P Ai C. Hijacking Antibody-Induced CTLA-4 Lysosomal Degradation for Safer and More Effective Cancer Immunotherapy. Cell Res (2019) 29:609–27. doi: 10.1038/s41422-019-0184-1 PMC6796842 31267017

[5] Krummel MF Bartumeus F Gerard A. T Cell Migration, Search Strategies and Mechanisms. Nat Rev Immunol (2016) 16:193–201. doi: 10.1038/nri.2015.16 26852928 PMC4869523

[6] Joyce JA Fearon DT. T Cell Exclusion, Immune Privilege, and the Tumor Microenvironment. Science (2015) 348:74–80. doi: 10.1126/science.aaa6204 25838376

[7] Rabinovich GA Gabrilovich D Sotomayor EM. Immunosuppressive Strategies That Are Mediated by Tumor Cells. Annu Rev Immunol (2007) 25:267–96. doi: 10.1146/annurev.immunol.25.022106.141609 PMC2895922 17134371

[8] Ishibashi K Kumai T Ohkuri T Kosaka A Nagato T Hirata Y. Epigenetic Modification Augments the Immunogenicity of Human Leukocyte Antigen G Serving as a Tumor Antigen for T Cell-Based Immunotherapy. Oncoimmunology (2016) 5:e1169356. doi: 10.1080/2162402X.2016.1169356 27471649 PMC4938368

[9] Terracina KP Graham LJ Payne KK Manjili MH Baek A Damle SR. DNA Methyltransferase Inhibition Increases Efficacy of Adoptive Cellular Immunotherapy of Murine Breast Cancer. Cancer Immunol Immunother (2016) 65:1061–73. doi: 10.1007/s00262-016-1868-8 PMC4996686 27416831

[10] Lucarini V Buccione C Ziccheddu G Peschiaroli F Sestili P Puglisi R. Combining Type I Interferons and 5-Aza-2’-Deoxycitidine to Improve Anti-Tumor Response Against Melanoma. J Invest Dermatol (2017) 137:159–69. doi: 10.1016/j.jid.2016.08.024 27623509

[11] Gollob JA Sciambi CJ. Decitabine Up-Regulates S100A2 Expression and Synergizes With IFN-Gamma to Kill Uveal Melanoma Cells. Clin Cancer Res (2007) 13:5219–25. doi: 10.1158/1078-0432.CCR-07-0816 17785578

[12] Krishnadas DK Shusterman S Bai F Diller L Sullivan JE Cheerva AC. A Phase I Trial Combining Decitabine/Dendritic Cell Vaccine Targeting MAGE-A1, MAGE-A3 and NY-ESO-1 for Children With Relapsed or Therapy-Refractory Neuroblastoma and Sarcoma. Cancer Immunol Immunother (2015) 64:1251–60. doi: 10.1007/s00262-015-1731-3 PMC11028635 26105625

[13] Leach DR Krummel MF Allison JP. Enhancement of Antitumor Immunity by CTLA-4 Blockade. Science (1996) 271:1734–6. doi: 10.1126/science.271.5256.1734 8596936

[14] Stamper CC Zhang Y Tobin JF Erbe DV Ikemizu S Davis SJ. Crystal Structure of the B7-1/CTLA-4 Complex That Inhibits Human Immune Responses. Nature (2001) 410:608–11. doi: 10.1038/35069118 11279502

[15] Iwai Y Ishida M Tanaka Y Okazaki T Honjo T Minato N. Involvement of PD-L1 on Tumor Cells in the Escape From Host Immune System and Tumor Immunotherapy by PD-L1 Blockade. Proc Natl Acad Sci USA (2002) 99:12293–7. doi: 10.1073/pnas.192461099 PMC129438 12218188

[16] Yamazaki T Akiba H Iwai H Matsuda H Aoki M Tanno Y. Expression of Programmed Death 1 Ligands by Murine T Cells and APC. J Immunol (2002) 169:5538–45. doi: 10.4049/jimmunol.169.10.5538 12421930

[17] Kuang DM Zhao Q Peng C Xu J Zhang JP Wu C. Activated Monocytes in Peritumoral Stroma of Hepatocellular Carcinoma Foster Immune Privilege and Disease Progression Through PD-L1. J Exp Med (2009) 206:1327–37. doi: 10.1084/jem.20082173 PMC2715058 19451266

[18] Pander J Heusinkveld M van der Straaten T Jordanova ES Baak-Pablo R Gelderblom H. Activation of Tumor-Promoting Type 2 Macrophages by EGFR-Targeting Antibody Cetuximab. Clin Cancer Res (2011) 17:5668–73. doi: 10.1158/1078-0432.CCR-11-0239 21788356

[19] Pages F Kirilovsky A Mlecnik B Asslaber M Tosolini M Bindea G. *In Situ* Cytotoxic and Memory T Cells Predict Outcome in Patients With Early-Stage Colorectal Cancer. J Clin Oncol (2009) 27:5944–51. doi: 10.1200/JCO.2008.19.6147 19858404

[20] Galon J Mlecnik B Bindea G Angell HK Berger A Lagorce C. Towards the Introduction of the ’Immunoscore’ in the Classification of Malignant Tumours. J Pathol (2014) 232:199–209. doi: 10.1002/path.4287 24122236 PMC4255306

[21] Lanitis E Dangaj D Irving M Coukos G. Mechanisms Regulating T-Cell Infiltration and Activity in Solid Tumors. Ann Oncol (2017) 28:xii18–32. doi: 10.1093/annonc/mdx238 29045511

[22] Peranzoni E Lemoine J Vimeux L Feuillet V Barrin S Kantari-Mimoun C. Macrophages Impede CD8 T Cells From Reaching Tumor Cells and Limit the Efficacy of Anti-PD-1 Treatment. Proc Natl Acad Sci USA (2018) 115:E4041–50. doi: 10.1073/pnas.1720948115 PMC5924916 29632196

[23] Shin JI Ha SJ. Regulatory T Cells-an Important Target for Cancer Immunotherapy. Nat Rev Clin Oncol (2014) 11:307. doi: 10.1038/nrclinonc.2013.208-c1 24781417

[24] Woo EY Chu CS Goletz TJ Schlienger K Yeh H Coukos G. Regulatory CD4(+)CD25(+) T Cells in Tumors From Patients With Early-Stage Non-Small Cell Lung Cancer and Late-Stage Ovarian Cancer. Cancer Res (2001) 61:4766–72. 11406550

[25] Togashi Y Shitara K Nishikawa H. Regulatory T Cells in Cancer Immunosuppression - Implications for Anticancer Therapy. Nat Rev Clin Oncol (2019) 16:356–71. doi: 10.1038/s41571-019-0175-7 30705439

[26] de Charette M Marabelle A Houot R. Turning Tumour Cells Into Antigen Presenting Cells: The Next Step to Improve Cancer Immunotherapy? Eur J Cancer (2016) 68:134–47. doi: 10.1016/j.ejca.2016.09.010 27755997

[27] Beatty GL Gladney WL. Immune Escape Mechanisms as a Guide for Cancer Immunotherapy. Clin Cancer Res (2015) 21:687–92. doi: 10.1158/1078-0432.CCR-14-1860 PMC4334715 25501578

[28] Dustin ML. The Immunological Synapse. Cancer Immunol Res (2014) 2:1023–33. doi: 10.1158/2326-6066.CIR-14-0161 PMC4692051 25367977

[29] Sigalotti L Fratta E Coral S Maio M. Epigenetic Drugs as Immunomodulators for Combination Therapies in Solid Tumors. Pharmacol Ther (2014) 142:339–50. doi: 10.1016/j.pharmthera.2013.12.015 24384533

[30] Heninger E Krueger TE Lang JM. Augmenting Antitumor Immune Responses With Epigenetic Modifying Agents. Front Immunol (2015) 6:29. doi: 10.3389/fimmu.2015.00029 25699047 PMC4316783

[31] Terranova-Barberio M Thomas S Munster PN. Epigenetic Modifiers in Immunotherapy: A Focus on Checkpoint Inhibitors. Immunotherapy (2016) 8:705–19. doi: 10.2217/imt-2016-0014 PMC5705793 27197539

[32] Maio M Covre A Fratta E Di Giacomo AM Taverna P Natali PG. Molecular Pathways: At the Crossroads of Cancer Epigenetics and Immunotherapy. Clin Cancer Res (2015) 21:4040–7. doi: 10.1158/1078-0432.CCR-14-2914 26374074

[33] Weintraub K. Take Two: Combining Immunotherapy With Epigenetic Drugs to Tackle Cancer. Nat Med (2016) 22:8–10. doi: 10.1038/nm0116-8 26735398

[34] Chiappinelli KB Zahnow CA Ahuja N Baylin SB. Combining Epigenetic and Immunotherapy to Combat Cancer. Cancer Res (2016) 76:1683–9. doi: 10.1158/0008-5472.CAN-15-2125 PMC4873370 26988985

[35] Jones PA Baylin SB. The Fundamental Role of Epigenetic Events in Cancer. Nat Rev Genet (2002) 3:415–28. doi: 10.1038/nrg816 12042769

[36] Esteller M. Epigenetics in Cancer. N Engl J Med (2008) 358:1148–59. doi: 10.1056/NEJMra072067 18337604

[37] Jones PA Takai D. The Role of DNA Methylation in Mammalian Epigenetics. Science (2001) 293:1068–70. doi: 10.1126/science.1063852 11498573

[38] Kouzarides T. Chromatin Modifications and Their Function. Cell (2007) 128:693–705. doi: 10.1016/j.cell.2007.02.005 17320507

[39] Jones PA Baylin SB. The Epigenomics of Cancer. Cell (2007) 128:683–92. doi: 10.1016/j.cell.2007.01.029 PMC3894624 17320506

[40] Shen H Laird PW. Interplay Between the Cancer Genome and Epigenome. Cell (2013) 153:38–55. doi: 10.1016/j.cell.2013.03.008 23540689 PMC3648790

[41] Li B Carey M Workman JL. The Role of Chromatin During Transcription. Cell (2007) 128:707–19. doi: 10.1016/j.cell.2007.01.015 17320508

[42] Fardi M Solali S Farshdousti Hagh M. Epigenetic Mechanisms as a New Approach in Cancer Treatment: An Updated Review. Genes Dis (2018) 5:304–11. doi: 10.1016/j.gendis.2018.06.003 PMC6303480 30591931

[43] Schubeler D. Function and Information Content of DNA Methylation. Nature (2015) 517:321–6. doi: 10.1038/nature14192 25592537

[44] Schotta G Lachner M Sarma K Ebert A Sengupta R Reuter G. A Silencing Pathway to Induce H3-K9 and H4-K20 Trimethylation at Constitutive Heterochromatin. Genes Dev (2004) 18:1251–62. doi: 10.1101/gad.300704 PMC420351 15145825

[45] Wang Z Zang C Rosenfeld JA Schones DE Barski A Cuddapah S. Combinatorial Patterns of Histone Acetylations and Methylations in the Human Genome. Nat Genet (2008) 40:897–903. doi: 10.1038/ng.154 18552846 PMC2769248

[46] Fraga MF Ballestar E Villar-Garea A Boix-Chornet M Espada J Schotta G. Loss of Acetylation at Lys16 and Trimethylation at Lys20 of Histone H4 Is a Common Hallmark of Human Cancer. Nat Genet (2005) 37:391–400. doi: 10.1038/ng1531 15765097

[47] Alexandrov LB Nik-Zainal S Wedge DC Aparicio SA Behjati S Biankin AV. Signatures of Mutational Processes in Human Cancer. Nature (2013) 500:415–21. doi: 10.1038/nature12477 PMC3776390 23945592

[48] Simpson AJ Caballero OL Jungbluth A Chen YT Old LJ. Cancer/testis Antigens, Gametogenesis and Cancer. Nat Rev Cancer (2005) 5:615–25. doi: 10.1038/nrc1669 16034368

[49] Whitehurst AW. Cause and Consequence of Cancer/Testis Antigen Activation in Cancer. Annu Rev Pharmacol Toxicol (2014) 54:251–72. doi: 10.1146/annurev-pharmtox-011112-140326 24160706

[50] Luo N Nixon MJ Gonzalez-Ericsson PI Sanchez V Opalenik SR Li H. DNA Methyltransferase Inhibition Upregulates MHC-I to Potentiate Cytotoxic T Lymphocyte Responses in Breast Cancer. Nat Commun (2018) 9:248. doi: 10.1038/s41467-017-02630-w 29339738 PMC5770411

[51] Magner WJ Kazim AL Stewart C Romano MA Catalano G Grande C. Activation of MHC Class I, II, and CD40 Gene Expression by Histone Deacetylase Inhibitors. J Immunol (2000) 165:7017–24. doi: 10.4049/jimmunol.165.12.7017 11120829

[52] Li H Chiappinelli KB Guzzetta AA Easwaran H Yen RW Vatapalli R. Immune Regulation by Low Doses of the DNA Methyltransferase Inhibitor 5-Azacitidine in Common Human Epithelial Cancers. Oncotarget (2014) 5:587–98. doi: 10.18632/oncotarget.1782 PMC3996658 24583822

[53] Ennishi D Takata K Beguelin W Duns G Mottok A Farinha P. Molecular and Genetic Characterization of MHC Deficiency Identifies EZH2 as Therapeutic Target for Enhancing Immune Recognition. Cancer Discov (2019) 9:546–63. doi: 10.1158/2159-8290.CD-18-1090 30705065

[54] Roulois D Loo Yau H Singhania R Wang Y Danesh A Shen SY. DNA-Demethylating Agents Target Colorectal Cancer Cells by Inducing Viral Mimicry by Endogenous Transcripts. Cell (2015) 162:961–73. doi: 10.1016/j.cell.2015.07.056 PMC4843502 26317465

[55] Chiappinelli KB Strissel PL Desrichard A Li H Henke C Akman B. Inhibiting DNA Methylation Causes an Interferon Response in Cancer *via* dsRNA Including Endogenous Retroviruses. Cell (2015) 162:974–86. doi: 10.1016/j.cell.2015.07.011 PMC4556003 26317466

[56] Heiland DH Haaker G Delev D Mercas B Masalha W Heynckes S. Comprehensive Analysis of PD-L1 Expression in Glioblastoma Multiforme. Oncotarget (2017) 8:42214–25. doi: 10.18632/oncotarget.15031 PMC5522061 28178682

[57] Gevensleben H Holmes EE Goltz D Dietrich J Sailer V Ellinger J. PD-L1 Promoter Methylation Is a Prognostic Biomarker for Biochemical Recurrence-Free Survival in Prostate Cancer Patients Following Radical Prostatectomy. Oncotarget (2016) 7:79943–55. doi: 10.18632/oncotarget.13161 PMC5346762 27835597

[58] Chen G Huang AC Zhang W Zhang G Wu M Xu W. Exosomal PD-L1 Contributes to Immunosuppression and Is Associated With Anti-PD-1 Response. Nature (2018) 560:382–6. doi: 10.1038/s41586-018-0392-8 PMC6095740 30089911

[59] Hogg SJ Vervoort SJ Deswal S Ott CJ Li J Cluse LA. BET-Bromodomain Inhibitors Engage the Host Immune System and Regulate Expression of the Immune Checkpoint Ligand PD-L1. Cell Rep (2017) 18:2162–74. doi: 10.1016/j.celrep.2017.02.011 PMC5340981 28249162

[60] Ebine K Kumar K Pham TN Shields MA Collier KA Shang M. Interplay Between Interferon Regulatory Factor 1 and BRD4 in the Regulation of PD-L1 in Pancreatic Stellate Cells. Sci Rep (2018) 8:13225. doi: 10.1038/s41598-018-31658-1 30185888 PMC6125340

[61] Zhu H Bengsch F Svoronos N Rutkowski MR Bitler BG Allegrezza MJ. BET Bromodomain Inhibition Promotes Anti-Tumor Immunity by Suppressing PD-L1 Expression. Cell Rep (2016) 16:2829–37. doi: 10.1016/j.celrep.2016.08.032 PMC5177024 27626654

[62] Kioussis D Georgopoulos K. Epigenetic Flexibility Underlying Lineage Choices in the Adaptive Immune System. Science (2007) 317:620–2. doi: 10.1126/science.1143777 17673651

[63] Ptaschinski C Mukherjee S Moore ML Albert M Helin K Kunkel SL. RSV-Induced H3K4 Demethylase KDM5B Leads to Regulation of Dendritic Cell-Derived Innate Cytokines and Exacerbates Pathogenesis *In Vivo* . PLoS Pathog (2015) 11:e1004978. doi: 10.1371/journal.ppat.1004978 26083387 PMC4470918

[64] Wilson CB Makar KW Perez-Melgosa M. Epigenetic Regulation of T Cell Fate and Function. J Infect Dis (2002) 185 Suppl 1:S37–45. doi: 10.1086/338001 11865438

[65] Zheng H Zhao W Yan C Watson CC Massengill M Xie M. And Augment Response to PD-1 Immunotherapy in Lung Adenocarcinoma. Clin Cancer Res (2016) 22:4119–32. doi: 10.1158/1078-0432.CCR-15-2584 PMC4987196 26964571

[66] Ghoneim HE Fan Y Moustaki A Abdelsamed HA Dash P Dogra P. *De Novo* Epigenetic Programs Inhibit PD-1 Blockade-Mediated T Cell Rejuvenation. Cell (2017) 170:142–57.e19. doi: 10.1016/j.cell.2017.06.007 28648661 PMC5568784

[67] Chang JT Wherry EJ Goldrath AW. Molecular Regulation of Effector and Memory T Cell Differentiation. Nat Immunol (2014) 15:1104–15. doi: 10.1038/ni.3031 PMC4386685 25396352

[68] Perez-Salvia M Esteller M. Bromodomain Inhibitors and Cancer Therapy: From Structures to Applications. Epigenetics (2017) 12:323–39. doi: 10.1080/15592294.2016.1265710 PMC5453193 27911230

[69] Xu Y Vakoc CR. Targeting Cancer Cells With BET Bromodomain Inhibitors. Cold Spring Harb Perspect Med 7 (2017) 7:7–25. doi: 10.1101/cshperspect.a026674 PMC5495050 28213432

[70] Youngblood B Hale JS Kissick HT Ahn E Xu X Wieland A. Effector CD8 T Cells Dedifferentiate Into Long-Lived Memory Cells. Nature (2017) 552:404–9. doi: 10.1038/nature25144 PMC5965677 29236683

[71] Carty SA Gohil M Banks LB Cotton RM Johnson ME Stelekati E. The Loss of TET2 Promotes CD8(+) T Cell Memory Differentiation. J Immunol (2018) 200:82–91. doi: 10.4049/jimmunol.1700559 29150566 PMC5736442

[72] Youngblood B Noto A Porichis F Akondy RS Ndhlovu ZM Austin JW. Cutting Edge: Prolonged Exposure to HIV Reinforces a Poised Epigenetic Program for PD-1 Expression in Virus-Specific CD8 T Cells. J Immunol (2013) 191:540–4. doi: 10.4049/jimmunol.1203161 PMC3702641 23772031

[73] Akondy RS Fitch M Edupuganti S Yang S Kissick HT Li KW. Origin and Differentiation of Human Memory CD8 T Cells After Vaccination. Nature (2017) 552:362–7. doi: 10.1038/nature24633 PMC6037316 29236685

[74] Nishikawa H Sakaguchi S. Regulatory T Cells in Cancer Immunotherapy. Curr Opin Immunol (2014) 27:1–7. doi: 10.1016/j.coi.2013.12.005 24413387

[75] Kitagawa Y Wing JB Sakaguchi S. Transcriptional and Epigenetic Control of Regulatory T Cell Development. Prog Mol Biol Transl Sci (2015) 136:1–33. doi: 10.1016/bs.pmbts.2015.07.011 26615090

[76] DuPage M Chopra G Quiros J Rosenthal WL Morar MM Holohan D. The Chromatin-Modifying Enzyme Ezh2 Is Critical for the Maintenance of Regulatory T Cell Identity After Activation. Immunity (2015) 42:227–38. doi: 10.1016/j.immuni.2015.01.007 PMC4347854 25680271

[77] Lee W Lee GR. Transcriptional Regulation and Development of Regulatory T Cells. Exp Mol Med (2018) 50:e456. doi: 10.1038/emm.2017.313 29520112 PMC5898904

[78] Morikawa H Sakaguchi S. Genetic and Epigenetic Basis of Treg Cell Development and Function: From a FoxP3-Centered View to an Epigenome-Defined View of Natural Treg Cells. Immunol Rev (2014) 259:192–205. doi: 10.1111/imr.12174 24712467

[79] Zorn E Nelson EA Mohseni M Porcheray F Kim H Litsa D. IL-2 Regulates FOXP3 Expression in Human CD4+CD25+ Regulatory T Cells Through a STAT-Dependent Mechanism and Induces the Expansion of These Cells *In Vivo* . Blood (2006) 108:1571–9. doi: 10.1182/blood-2006-02-004747 PMC1895505 16645171

[80] Floess S Freyer J Siewert C Baron U Olek S Polansky J. Epigenetic Control of the Foxp3 Locus in Regulatory T Cells. PLoS Biol (2007) 5:e38. doi: 10.1371/journal.pbio.0050038 17298177 PMC1783672

[81] Lal G Bromberg JS. Epigenetic Mechanisms of Regulation of Foxp3 Expression. Blood (2009) 114:3727–35. doi: 10.1182/blood-2009-05-219584 PMC2773485 19641188

[82] Zhou L Chong MM Littman DR. Plasticity of CD4+ T Cell Lineage Differentiation. Immunity (2009) 30:646–55. doi: 10.1016/j.immuni.2009.05.001 19464987

[83] Russ BE Prier JE Rao S Turner SJ. T Cell Immunity as a Tool for Studying Epigenetic Regulation of Cellular Differentiation. Front Genet (2013) 4:218. doi: 10.3389/fgene.2013.00218 24273551 PMC3824109

[84] Srivastava MK Sinha P Clements VK Rodriguez P Ostrand-Rosenberg S. Myeloid-Derived Suppressor Cells Inhibit T-Cell Activation by Depleting Cystine and Cysteine. Cancer Res (2010) 70:68–77. doi: 10.1158/0008-5472.CAN-09-2587 20028852 PMC2805057

[85] Zhang H Li ZL Ye SB Ouyang LY Chen YS He J. Myeloid-Derived Suppressor Cells Inhibit T Cell Proliferation in Human Extranodal NK/T Cell Lymphoma: A Novel Prognostic Indicator. Cancer Immunol Immunother (2015) 64:1587–99. doi: 10.1007/s00262-015-1765-6 PMC4643115 26497849

[86] Nagaraj S Schrum AG Cho HI Celis E Gabrilovich DI. Mechanism of T Cell Tolerance Induced by Myeloid-Derived Suppressor Cells. J Immunol (2010) 184:3106–16. doi: 10.4049/jimmunol.0902661 PMC2832724 20142361

[87] Alvarez-Errico D Vento-Tormo R Sieweke M Ballestar E. Epigenetic Control of Myeloid Cell Differentiation, Identity and Function. Nat Rev Immunol (2015) 15:7–17. doi: 10.1038/nri3777 25534619

[88] Ivashkiv LB Park SH. Epigenetic Regulation of Myeloid Cells. Microbiol Spectr (2016) 4:571–90. doi: 10.1128/microbiolspec.MCHD-0010-2015 PMC5268423 27337441

[89] Sahakian E Powers JJ Chen J Deng SL Cheng F Distler A. Histone Deacetylase 11: A Novel Epigenetic Regulator of Myeloid Derived Suppressor Cell Expansion and Function. Mol Immunol (2015) 63:579–85. doi: 10.1016/j.molimm.2014.08.002 PMC4252813 25155994

[90] Zhang C Wang S Liu Y Yang C. Epigenetics in Myeloid Derived Suppressor Cells: A Sheathed Sword Towards Cancer. Oncotarget (2016) 7:57452–63. doi: 10.18632/oncotarget.10767 PMC5303001 27458169

[91] Dai Z Ramesh V Locasale JW. The Evolving Metabolic Landscape of Chromatin Biology and Epigenetics. Nat Rev Genet (2020) 21:737–53. doi: 10.1038/s41576-020-0270-8 PMC8059378 32908249

[92] Faubert B Solmonson A DeBerardinis RJ. Metabolic Reprogramming and Cancer Progression. Science (2020) 368:eaaw5473. doi: 10.1126/science.aaw5473 32273439 PMC7227780

[93] Zheng Q Maksimovic I Upad A David Y. Non-Enzymatic Covalent Modifications: A New Link Between Metabolism and Epigenetics. Protein Cell (2020) 11:401–16. doi: 10.1007/s13238-020-00722-w PMC7251012 32356279

[94] Wang YP Lei QY. Metabolic Recoding of Epigenetics in Cancer. Cancer Commun (Lond) (2018) 38:25. doi: 10.1186/s40880-018-0302-3 29784032 PMC5993135

[95] Thakur C Chen F. Connections Between Metabolism and Epigenetics in Cancers. Semin Cancer Biol (2019) 57:52–8. doi: 10.1016/j.semcancer.2019.06.006 PMC6690786 31185282

[96] Varier RA Timmers HT. Histone Lysine Methylation and Demethylation Pathways in Cancer. Biochim Biophys Acta (2011) 1815:75–89. doi: 10.1016/j.bbcan.2010.10.002 20951770

[97] Klose RJ Kallin EM Zhang Y. JmjC-Domain-Containing Proteins and Histone Demethylation. Nat Rev Genet (2006) 7:715–27. doi: 10.1038/nrg1945 16983801

[98] Desai S Ding M Wang B Lu Z Zhao Q Shaw K. Tissue-Specific Isoform Switch and DNA Hypomethylation of the Pyruvate Kinase PKM Gene in Human Cancers. Oncotarget (2014) 5:8202–10. doi: 10.18632/oncotarget.1159 PMC4226677 24077665

[99] Kelly B O’Neill LA. Metabolic Reprogramming in Macrophages and Dendritic Cells in Innate Immunity. Cell Res (2015) 25:771–84. doi: 10.1038/cr.2015.68 PMC4493277 26045163

[100] Shyer JA Flavell RA Bailis W. Metabolic Signaling in T Cells. Cell Res (2020) 30:649–59. doi: 10.1038/s41422-020-0379-5 PMC7395146 32709897

[101] Bian Y Li W Kremer DM Sajjakulnukit P Li S Crespo J. Cancer SLC43A2 Alters T Cell Methionine Metabolism and Histone Methylation. Nature (2020) 585:277–82. doi: 10.1038/s41586-020-2682-1 PMC7486248 32879489

[102] Puleston DJ Baixauli F Sanin DE Edwards-Hicks J Villa M Kabat AM. Polyamine Metabolism Is a Central Determinant of Helper T Cell Lineage Fidelity. Cell (2021) 184:4186–202.e20. doi: 10.1016/j.cell.2021.06.007 34216540 PMC8358979

[103] Franco F Jaccard A Romero P Yu YR Ho PC. Metabolic and Epigenetic Regulation of T-Cell Exhaustion. Nat Metab (2020) 2:1001–12. doi: 10.1038/s42255-020-00280-9 32958939

[104] Larkin J Chiarion-Sileni V Gonzalez R Grob JJ Cowey CL Lao CD. Combined Nivolumab and Ipilimumab or Monotherapy in Untreated Melanoma. N Engl J Med (2015) 373:23–34. doi: 10.1056/NEJMoa1504030 26027431 PMC5698905

[105] Topper MJ Vaz M Chiappinelli KB DeStefano Shields CE Niknafs N Yen RC. Epigenetic Therapy Ties MYC Depletion to Reversing Immune Evasion and Treating Lung Cancer. Cell (2017) 171:1284–300.e21. doi: 10.1016/j.cell.2017.10.022 29195073 PMC5808406

[106] Fukumoto T Fatkhutdinov N Zundell JA Tcyganov EN Nacarelli T Karakashev S. HDAC6 Inhibition Synergizes With Anti-PD-L1 Therapy in ARID1A-Inactivated Ovarian Cancer. Cancer Res (2019) 79:5482–9. doi: 10.1158/0008-5472.CAN-19-1302 PMC6825538 31311810

[107] Knox T Sahakian E Banik D Hadley M Palmer E Noonepalle S. Selective HDAC6 Inhibitors Improve Anti-PD-1 Immune Checkpoint Blockade Therapy by Decreasing the Anti-Inflammatory Phenotype of Macrophages and Down-Regulation of Immunosuppressive Proteins in Tumor Cells. Sci Rep (2019) 9:6136. doi: 10.1038/s41598-019-42237-3 30992475 PMC6467894

[108] Fratta E Coral S Covre A Parisi G Colizzi F Danielli R. The Biology of Cancer Testis Antigens: Putative Function, Regulation and Therapeutic Potential. Mol Oncol (2011) 5:164–82. doi: 10.1016/j.molonc.2011.02.001 PMC5528287 21376678

[109] James SR Link PA Karpf AR. Epigenetic Regulation of X-Linked Cancer/Germline Antigen Genes by DNMT1 and DNMT3b. Oncogene (2006) 25:6975–85. doi: 10.1038/sj.onc.1209678 16715135

[110] Weber J Salgaller M Samid D Johnson B Herlyn M Lassam N. Expression of the MAGE-1 Tumor Antigen Is Up-Regulated by the Demethylating Agent 5-Aza-2’-Deoxycytidine. Cancer Res (1994) 54:1766–71. 7511051

[111] Luo W Wang X Kageshita T Wakasugi S Karpf AR Ferrone S. Regulation of High Molecular Weight-Melanoma Associated Antigen (HMW-MAA) Gene Expression by Promoter DNA Methylation in Human Melanoma Cells. Oncogene (2006) 25:2873–84. doi: 10.1038/sj.onc.1209319 16407841

[112] Wischnewski F Pantel K Schwarzenbach H. Promoter Demethylation and Histone Acetylation Mediate Gene Expression of MAGE-A1, -A2, -A3, and -A12 in Human Cancer Cells. Mol Cancer Res (2006) 4:339–49. doi: 10.1158/1541-7786.MCR-05-0229 16687489

[113] Ritter C Fan K Paschen A Reker Hardrup S Ferrone S Nghiem P. Epigenetic Priming Restores the HLA Class-I Antigen Processing Machinery Expression in Merkel Cell Carcinoma. Sci Rep (2017) 7:2290. doi: 10.1038/s41598-017-02608-0 28536458 PMC5442125

[114] Khan AN Gregorie CJ Tomasi TB. Histone Deacetylase Inhibitors Induce TAP, LMP, Tapasin Genes and MHC Class I Antigen Presentation by Melanoma Cells. Cancer Immunol Immunother (2008) 57:647–54. doi: 10.1007/s00262-007-0402-4 PMC3146348 18046553

[115] Kitamura H Torigoe T Asanuma H Honma I Sato N Tsukamoto T. Down-Regulation of HLA Class I Antigens in Prostate Cancer Tissues and Up-Regulation by Histone Deacetylase Inhibition. J Urol (2007) 178:692–6. doi: 10.1016/j.juro.2007.03.109 17574613

[116] Sheng W LaFleur MW Nguyen TH Chen S Chakravarthy A Conway JR. LSD1 Ablation Stimulates Anti-Tumor Immunity and Enables Checkpoint Blockade. Cell (2018) 174:549–63.e19. doi: 10.1016/j.cell.2018.05.052 29937226 PMC6063761

[117] Wherry EJ Kurachi M. Molecular and Cellular Insights Into T Cell Exhaustion. Nat Rev Immunol (2015) 15:486–99. doi: 10.1038/nri3862 PMC4889009 26205583

[118] Pauken KE Sammons MA Odorizzi PM Manne S Godec J Khan O. Epigenetic Stability of Exhausted T Cells Limits Durability of Reinvigoration by PD-1 Blockade. Science (2016) 354:1160–5. doi: 10.1126/science.aaf2807 PMC5484795 27789795

[119] Zhang F Zhou X DiSpirito JR Wang C Wang Y Shen H. Epigenetic Manipulation Restores Functions of Defective CD8(+) T Cells From Chronic Viral Infection. Mol Ther (2014) 22:1698–706. doi: 10.1038/mt.2014.91 PMC4435497 24861055

[120] Woods DM Sodre AL Villagra A Sarnaik A Sotomayor EM Weber J. HDAC Inhibition Upregulates PD-1 Ligands in Melanoma and Augments Immunotherapy With PD-1 Blockade. Cancer Immunol Res (2015) 3:1375–85. doi: 10.1158/2326-6066.CIR-15-0077-T PMC4674300 26297712

[121] Peng D Kryczek I Nagarsheth N Zhao L Wei S Wang W. Epigenetic Silencing of TH1-Type Chemokines Shapes Tumour Immunity and Immunotherapy. Nature (2015) 527:249–53. doi: 10.1038/nature15520 PMC4779053 26503055

[122] Zhu S Denman CJ Cobanoglu ZS Kiany S Lau CC Gottschalk SM. The Narrow-Spectrum HDAC Inhibitor Entinostat Enhances NKG2D Expression Without NK Cell Toxicity, Leading to Enhanced Recognition of Cancer Cells. Pharm Res (2015) 32:779–92. doi: 10.1007/s11095-013-1231-0 PMC4014531 24203492

[123] Ahearne MJ Allchin RL Fox CP Wagner SD. Follicular Helper T-Cells: Expanding Roles in T-Cell Lymphoma and Targets for Treatment. Br J Haematol (2014) 166:326–35. doi: 10.1111/bjh.12941 24815671

[124] Lopez-Soto A Folgueras AR Seto E Gonzalez S. HDAC3 Represses the Expression of NKG2D Ligands ULBPs in Epithelial Tumour Cells: Potential Implications for the Immunosurveillance of Cancer. Oncogene (2009) 28:2370–82. doi: 10.1038/onc.2009.117 19430493

[125] Yamanegi K Yamane J Kobayashi K Kato-Kogoe N Ohyama H Nakasho K. Valproic Acid Cooperates With Hydralazine to Augment the Susceptibility of Human Osteosarcoma Cells to Fas- and NK Cell-Mediated Cell Death. Int J Oncol (2012) 41:83–91. doi: 10.3892/ijo.2012.1438 22576685

[126] Wang L Amoozgar Z Huang J Saleh MH Xing D Orsulic S. Decitabine Enhances Lymphocyte Migration and Function and Synergizes With CTLA-4 Blockade in a Murine Ovarian Cancer Model. Cancer Immunol Res (2015) 3:1030–41. doi: 10.1158/2326-6066.CIR-15-0073 26056145

[127] Yu G Wu Y Wang W Xu J Lv X Cao X. Low-Dose Decitabine Enhances the Effect of PD-1 Blockade in Colorectal Cancer With Microsatellite Stability by Re-Modulating the Tumor Microenvironment. Cell Mol Immunol (2019) 16:401–9. doi: 10.1038/s41423-018-0026-y PMC6461874 29622799

[128] Wrangle J Wang W Koch A Easwaran H Mohammad HP Vendetti F. Alterations of Immune Response of Non-Small Cell Lung Cancer With Azacytidine. Oncotarget (2013) 4:2067–79. doi: 10.18632/oncotarget.1542 PMC3875770 24162015

[129] West AC Smyth MJ Johnstone RW. The Anticancer Effects of HDAC Inhibitors Require the Immune System. Oncoimmunology (2014) 3:e27414. doi: 10.4161/onci.27414 24701376 PMC3962507

[130] Oki Y Buglio D Zhang J Ying Y Zhou S Sureda A. Immune Regulatory Effects of Panobinostat in Patients With Hodgkin Lymphoma Through Modulation of Serum Cytokine Levels and T-Cell PD1 Expression. Blood Cancer J (2014) 4:e236. doi: 10.1038/bcj.2014.58 25105535 PMC4219471

[131] Kim K Skora AD Li Z Liu Q Tam AJ Blosser RL. Eradication of Metastatic Mouse Cancers Resistant to Immune Checkpoint Blockade by Suppression of Myeloid-Derived Cells. Proc Natl Acad Sci USA (2014) 111:11774–9. doi: 10.1073/pnas.1410626111 PMC4136565 25071169

[132] Orillion A Hashimoto A Damayanti N Shen L Adelaiye-Ogala R Arisa S. Entinostat Neutralizes Myeloid-Derived Suppressor Cells and Enhances the Antitumor Effect of PD-1 Inhibition in Murine Models of Lung and Renal Cell Carcinoma. Clin Cancer Res (2017) 23:5187–201. doi: 10.1158/1078-0432.CCR-17-0741 PMC5723438 28698201

[133] Leslie M. First EZH2 Inhibitor Approved-For Rare Sarcoma. Cancer Discov (2020) 10:333–4. doi: 10.1158/2159-8290.CD-NB2020-006 32041739

[134] Goswami S Apostolou I Zhang J Skepner J Anandhan S Zhang X. Modulation of EZH2 Expression in T Cells Improves Efficacy of Anti-CTLA-4 Therapy. J Clin Invest (2018) 128:3813–8. doi: 10.1172/JCI99760 PMC6118570 29905573

